# Care Cascade for targeted tuberculosis testing and linkage to Care in Homeless Populations in the United States: a meta-analysis

**DOI:** 10.1186/s12889-018-5393-x

**Published:** 2018-04-12

**Authors:** Andrea Parriott, Mohsen Malekinejad, Amanda P. Miller, Suzanne M. Marks, Hacsi Horvath, James G. Kahn

**Affiliations:** 1 Philip R. Lee Institute for Health Policy Studies, University of California, San Francisco, 3333 California St., Ste. 265, Box 0936, San Francisco, CA 94118 USA; 2 Division of Tuberculosis Elimination, Centers for Disease Control and Prevention, Mailstop E-10, 1600 Clifton Road, Atlanta, GA 30333 USA

**Keywords:** Homelessness, Tuberculosis, Targeted testing, United States, Health disparities, Health services, Adults, Public health

## Abstract

**Background:**

Homelessness increases the risk of tuberculosis (TB) disease and latent TB infection (LTBI), but persons experiencing homelessness often lack access to testing and treatment. We assessed the yield of TB testing and linkage to care for programs targeting homeless populations in the United States.

**Methods:**

We conducted a comprehensive search of peer-reviewed and grey literature, adapting Cochrane systematic review methods. Two reviewers independently assessed study eligibility and abstracted key data on the testing to care cascade: number of persons reached, recruited for testing, tested for LTBI, with valid test results, referred to follow-up care, and initiating care. We used random effects to calculate pooled proportions and 95% confidence intervals (CI) of persons retained in each step via inverse-variance weighted meta-analysis, and cumulative proportions as products of adjacent step proportions.

**Results:**

We identified 23 studies published between 1986 and 2014, conducted in 12 states and 15 cities. Among studies using tuberculin skin tests (TST) we found that 93.7% (CI 72.4-100%) of persons reached were recruited, 97.9% (89.3-100%) of those recruited had tests placed, 85.5% (78.6-91.3%) of those with tests placed returned for reading, 99.9% (99.6-100%) of those with tests read had valid results, and 24.7% (21.0-28.5%) with valid results tested positive. All persons testing positive were referred to follow-up care, and 99.8% attended at least one session of follow-up care. Heterogeneity was high for most pooled proportions. For a hypothetical cohort of 1000 persons experiencing homelessness reached by a targeted testing program using TST, an estimated 917 were tested, 194 were positive, and all of these initiated follow-up care.

**Conclusions:**

Targeted TB testing of persons experiencing homelessness appears effective in detecting LTBI and connecting persons to care and potential treatment. Future evaluations should assess diagnostic use of interferon gamma release assays and completion of treatment, and costs of testing and treatment.

**Electronic supplementary material:**

The online version of this article (10.1186/s12889-018-5393-x) contains supplementary material, which is available to authorized users.

## Background

Nearly 600,000 people in the United States (US) experienced homelessness on any one night in 2015, whether they slept in homeless shelters or in other locations [1]. Homelessness is a high risk factor for tuberculosis disease (TB) and latent TB infection (LTBI) [2] and persons experiencing homelessness are at an estimated 10 times greater risk of being diagnosed with TB disease than the general population [3]. More than 5% of TB cases reported to the National TB Surveillance System have experienced homelessness in the preceding year [4]. Homelessness increases risk of TB due to exposure in crowded shelters, and its association with substance use, and HIV infection, which lowers immunity [2].

It is critical to diagnose LTBI in patients in order to provide treatment, prevent disease progression, and subsequently prevent TB transmission should progression to TB disease occur. Early TB infection diagnosis in homeless populations can be challenging because many have barriers to accessing health care, including lack of health insurance, difficulty paying for care, lack of transportation, and lack of information needed to access care [2, 5, 6]. Linking homeless persons diagnosed with LTBI to further follow-up and assessment for treatment is critical for their health and for TB disease elimination efforts. Targeted tuberculosis testing programs (TTTs) test persons from high risk populations, such as persons experiencing homelessness or persons born in high incidence countries, for LTBI. This is done using one of two test types, the tuberculin skin test (TST) or an interferon gamma release assay (IGRA). There are advantages and disadvantages to each test type in homeless populations. TSTs require that persons who have tests placed, return within 48 to 72 h to have the test read. Coming back to the test site within a fixed time period can be challenging for persons experiencing homelessness who may relocate frequently, are financially stressed, and commonly suffer from mental health disorders. In contrast, IGRAs are performed in a lab, and do not require any contact with the person being tested, aside from the initial blood draw, to obtain valid test results. However, persons who test positive must be located in order to inform them of their test results and coordinate follow-up care, which can be very challenging for persons without fixed addresses or contact information, whereas with TSTs, positive results can be communicated and follow-up referrals given immediately once the person returns for TST reading.

Strategies for increasing targeted testing for LTBI and linkage to care of homeless populations may help to decrease TB incidence. In this review, we seek to assess the yield of such programs by assessing the proportion of persons experiencing homelessness who are retained in each step of the TTT cascade.

## Methods

Our search, screening, study selection and analysis methods are based on those of the Cochrane Collaboration, as presented in the Cochrane Handbook for Systematic Reviews of Interventions [7]. We followed the Preferred Reporting Items for Systematic Reviews and Meta-analyses (PRISMA) guidance [8] in reporting our review. Our protocol is registered on PROSPERO (CRD42016039432) [9].

### Eligibility criteria

We included studies reporting the results of TTT programs in adolescents and adults experiencing homelessness in the US. Programs needed to target homeless populations specifically, but could include a minority of participants who were stably housed. We defined study populations as homeless if participants were recruited from homeless shelters, health care clinics for homeless populations, or other service agencies primarily serving homeless clientele. We also included studies that described the study populations as homeless or used related terms such as unhoused, unstably housed, itinerant, street youth or other such terms (see Additional file 1).

Eligible studies must have used a biological test such as tuberculin skin test (TST) or interferon gamma release assay (IGRA) to ascertain LTBI. Eligible studies needed to report, at minimum, the numbers of participants with valid LTBI test results and numbers of those testing positive. We excluded studies that used only self-report to assess LTBI, as well as contact investigations, studies where stored specimens were tested for evidence of infection, and studies reporting testing results unlinked to patient identity. We excluded studies that tested participants only for active TB disease (i.e., not LTBI), such as those reporting only data from chest radiographs or sputum testing. We also excluded studies that primarily focused on populations aged 14 or under.

We did not have any eligibility restrictions based on publication status, study design, or language of publication. Single arm and multi-arm studies were eligible. Studies from peer reviewed literature or from conference abstracts and other grey literature were eligible for inclusion.

### Search, screening, and study selection

We developed a comprehensive search strategy that included multiple variations and synonyms of relevant TB and “homelessness” terms as well as National Library of Medicine Medical Subject Heading (MeSH) terms and Embase “Emtree” indexing terms. Because our original conception of this review also included studies addressing HIV, hepatitis B and hepatitis C in homeless populations, our strategy includes terms relevant to those conditions. We subsequently decided to report our review of studies concerned with these blood-borne infections in a separate manuscript [data analysis report provided to the US Centers for Disease Control and Prevention (CDC) on September 21, 2016]. In this review we focus only on TB studies.

We searched PubMed, Embase, Web of Science and the Cochrane Central Register of Controlled Trials, from earliest records to June 13, 2016. Additional file 1 provides our search strategies. We additionally hand-searched CDC’s TB Notes Newsletters and available abstracts from the National TB Controllers’ Association National TB Conferences and searched abstracts from the American Public Health Association’s annual conferences. We reviewed the bibliographies of our included studies as well as references cited in a previous systematic review which examined the prevalence of TB disease in homeless populations [10].

Two persons, working independently, applied eligibility criteria to titles and abstracts of all studies captured in the searches and each identified a selection of potentially-eligible studies. They then compared their respective selections and reached consensus about potential eligibility. A third author stood ready to serve as neutral arbiter in case consensus was not reached, but this was never necessary. Two persons then reviewed full-text articles of records deemed potentially eligible and in an identical process, made final decisions about study eligibility.

### Elements of the testing and care Cascade

Outputs of interest in the TTT cascade were the number of participants who:

1. Were Reached: contacted and invited to participate in the testing program.
2. Were Recruited: agreed to participate in the testing program.
3. Were Tested: had TSTs placed or blood drawn for IGRA testing.
4. Had a TST read: had a TST evaluated. This step does not apply to studies that used IGRA.
5. Had valid results: with a clear positive or negative test result given to testing program staff. This excludes participants with inconclusive IGRA or TST results.
6. Tested positive: tested positive for TB infection. We used the respective studies’ definitions of positive test results.
7. Were referred for follow-up: provided with information or an appointment to receive further evaluation of positive test results and treatment, if appropriate.
8. Attended follow-up: attended at least one session for evaluation of a positive test and/or for treatment services. This includes participants who were evaluated for LTBI treatment, but deemed to be unsuitable candidates for therapy.

We also extracted the number of active cases that were diagnosed upon follow-up of positive TSTs or IGRAs, but did not consider it part of the “cascade” because our cascade ends with attendance for follow-up care, which must occur prior to TB disease or LTBI diagnosis. As this review focuses on testing for LTBI, cases of TB disease diagnosed by chest X-ray or sputum sample done in the absence of a positive TST or IGRA, were not extracted.

### Data extraction

One person extracted key data into a pre-piloted and standardized data extraction spreadsheet (Additional file 2). A second person independently extracted data from the TTT care cascade, blinded to the first author’s extraction, and checked the remainder of the first extraction for accuracy. These independent extractions were compared and reconciled via consensus or by decision of the arbiter.

### Risk of bias assessment

We did not formally assess risk of bias in the individual studies since existing standard instruments for assessing bias risk for intervention efficacy, prevalence, and other epidemiologic studies were not applicable to studies reporting yield of screening programs.

### Statistical analysis and data synthesis

All analyses were conducted using Stata version 14. Proportions of persons proceeding from one step in the testing and linkage to care cascade to subsequent steps and associated 95% confidence intervals (CIs) were calculated using the Wald method. As we wanted to assess the performance of these programs in detecting new cases of LTBI, when studies reported testing persons with a known history of TB infection or TB disease, we subtracted their number from all cascade steps except the number reached. If two or more studies reported data for the same proportion, pooled proportions were calculated using inverse-variance random effects meta-analysis. The Freeman-Tukey double arcsine transformation was used to normalize individual study proportions prior to pooling. Where data existed, we also analyzed proportions stratified by recruitment method (health-care facility based recruitment vs. recruitment from other service agencies).

We calculated pooled cumulative proportions for each step in the cascade for the subset of studies that used TST for testing. Cumulative proportions were products of adjacent-step pooled proportions. The cumulative proportion tested of those reached was equal to the product of the proportion recruited of those reached and the proportion tested of those recruited. The cumulative proportion with valid test results of those reached was equal to the product of the cumulative proportion tested of those reached and the proportion with valid test results of those tested. Each subsequent cumulative proportion was the product of a single step proportion and the cumulative proportion that immediately proceeded it. We also calculated cumulative proportions stratified by test type, using test specific values for the proportion with valid test results of those tested and the proportion testing positive of those with valid test results. Confidence intervals for cumulative proportions were calculated using a simulation method. Each pooled proportion and confidence limit closest to 0.5 were normalized using the Freeman-Tukey double arcsine transformation, with a sample size equal to the harmonic mean of the individual study sample sizes and the number of successes equal to the product of the pooled proportion and the harmonic mean. For each pooled proportion, we took 50,000 draws from a normal distribution with a mean equal to the transformed pooled proportion and a standard deviation equal to the absolute value of the difference between the transformed mean and the transformed confidence limit divided by 1.96. Draws less than the transformed value of zero were replaced with the transformed value of zero, and draws greater than the transformed value of one were replaced with the transformed value of one. Draws were then reverse transformed into proportions to build probability distributions for each pooled proportions. Draws for each proportion were then multiplied with draws from the next proportion in the cascade in a sequential manner, and the 95% confidence intervals were taken from the 2.5th and 97.5th percentile of the resulting product distributions.

The time trend for the proportions testing positive of persons with valid results for studies using TST that included dates of data collection was analyzed using the Spearman’s rank correlation coefficient.

## Results

### Identified studies

**Fig. 1:**
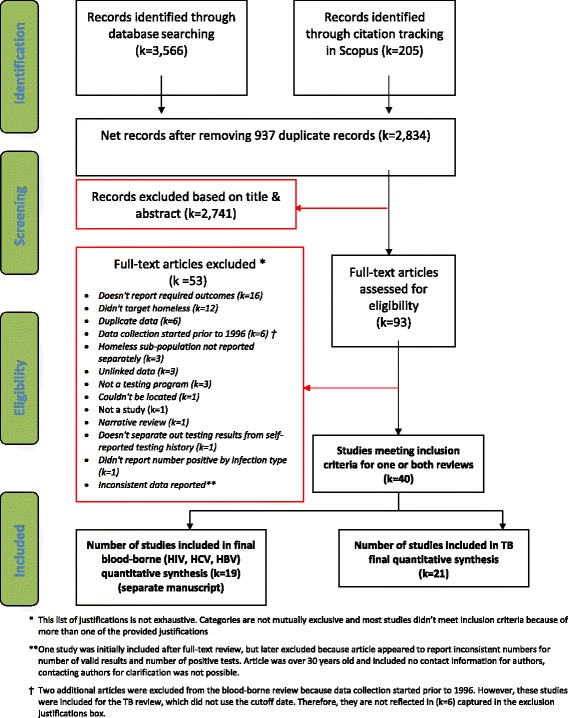
PRISMA flowchart. Process of identification and screening of citations from article databases, targeted testing and linkage to care studies among homeless populations in the United States

From a total of 3566 peer-reviewed citations identified through article databases and tracking of cited references of included studies in Scopus, 40 met inclusion criteria for either the TB or the blood-borne disease review, 21 of these reported outcomes of TB testing. Figure 1 details this process, and Additional file 3 lists reasons for exclusion after full-text review. In addition to 21 indexed publications, we found one eligible study cited in CDC’s peer-reviewed newsletter “TB Notes” and one conference abstract via our grey literature search. Descriptions of the included studies are in Table 1. Studies were conducted in 12 states and at least 15 cities (including six in New York City). With the possible exception of one study conducted in an unnamed county in Indiana [11], all studies took place in urban areas. Studies were published or presented between 1986 and 2014, with data collection reported to be between 1982 and 2009. Of 15 studies [12–26] that reported gender distribution, 8 [12, 14, 17, 18, 21, 24–26] had exclusively male study populations, the remainder had between 4% and 22% female study populations. In most studies that reported race, the majority of the populations were African American. Two studies used IGRA [27, 28], and one study used a combination of TST and IGRA [11] for testing, the remainder relied exclusively on TST.

**Table 1:** Characteristics of 23 TB targeted testing and linkage to care studies among homeless populations in the United States

Author and year	Data collection years	City/county, State	Target population	Recruitment method	Number of valid test results
Barry 1986 [15]	1984	Boston, Massachusetts	Homeless adults	Shelter-based	187
McAdam 1990 [15]	1982-1988	New York, New York	Homeless adults	Shelter-based	1508
Torres 1990 [16]	1986-1989	New York, New York	Individuals staying at a “Gay Mens” Shelter on Ward Island	Shelter, and healthcare facility-based	94
Nolan 1991 [17]	1985	Seattle, Washington	Homeless men	Shelter-based	93
Neims 1992 [18]	Not reported	Baltimore, Maryland	Individuals who spent at least one night in a homeless shelter in the previous year.	Shelter, healthcare facility, and other service-based	57
Paul 1993 [20]	1990	New York, New York	Homeless men	Shelter-based	98
Gelberg 1997 [21]	1991 or 1992- not reported	Los Angeles, California	Homeless adults	Participants were recruited from a separate longitudinal cohort of homeless persons conducted by the RAND Corporation; the RAND cohort was recruited using a combination of shelter-based, meal program-based, and street-based recruitment	260
Morrow 1997 [22]	Not reported	Yonkers, New York	Underserved men and women using the shelter’s services (food, clinic, sleeping there)	Shelter-based	95
Sakai 1998 [19]	1996	New Orleans, Louisiana	Homeless men	Shelter-based	105
Bock 1999 [23]	1994-1996	Atlanta, Georgia	“High- risk” inner city residents	Healthcare facility-based	2002
Griffin 1999 [24]	1997-1998	Kansasa City, Missouri	Homeless adults	Shelter-based	654
Kimerling 1999 [25]	1996-1996	Birmingham, Alabama	Homeless adults	Shelter-based	21
Falchook 2000 [26]	1998-1999	New Orleans, Louisiana	Homeless adults	Shelter-based	54
Kong 2002 [27]	1995-1998	Denver, Colorado	Residents of communal shelters and residential drug and alcohol treatment programs	Shelter and other service-based	10,207
Cheung 2002 [28]	1995-2000	Menlo Park, California	VA-eligible homeless adults	Healthcare facility-based	829
Saez 2002 [32]	1990-1992	New York City, New York	Mentally ill homeless men	Shelter and healthcare facility-based	75
Forman 2003 [29]	2001	Anchorage, Alaska	Homeless adults	Shelter-based	47
Valencia 2004 [30]	Not reported	New York, New York	Mentally ill homeless men	Shelter-based	173
Dewan 2006 [12]	2003-2005	San Francisco, California	Homeless adults	Healthcare facility-based	2559
Lashley 2007 [31]	2005-2007	Baltimore, Maryland	Homeless adults in or recently graduated from a residential addictions recovery program	Shelter-based	282
McAdam 2009 [33]	1992-2006	New York, New York	Homeless persons attending shelters and drop-in facilities	Shelter and other service-based	21,028
Alexander 2011 [11]	2009-2011	County not reported, Indiana	Homeless adults	Shelter-based with street outreach component: health fairs and another event offering legal, social and health services to the homeless.	1421
Higashi 2014 [13]	2005-2009	San Francisco, California	Homeless adults	Healthcare facility-based	10,935

### Test and linkage to care cascade – TST studies

Twenty studies [12–26, 29–34] used TST for testing. Additional file 4: Table S1 reports proportions of persons proceeding from each cascade step to subsequent steps, and the number of studies contributing data on each proportion. Of persons reached, 93.7% (95% CI 72.4 to 100%) were recruited; of persons recruited, 97.9% (89.3 to 100%) had tests placed; of those with tests placed, 85.5% (78.6 to 91.3%) returned to have tests read; of those with tests read, 99.9% (99.6 to 100%) had valid test results; and of those with valid test results, 24.7% (21.0 to 28.5%) tested positive. Of four studies [12, 14, 19, 26] that reported number given a referral for follow-up, all persons who tested positive were referred, and 99.8% of persons who agreed to be referred to care attended at least once. Among the eleven studies [12, 14, 15, 19, 21, 23, 25, 29, 31–33] that reported number of TB disease cases diagnosed among persons tested for TB infection, 1.2% (0.0 to 3.4%) of persons testing positive were diagnosed with TB disease. The proportion of variability in the effect estimates that is due to heterogeneity (I^2^) was greater than 75% for all proportions where I^2^ was calculable, except the proportion referred to treatment of those testing positive (I^2^ = 0), for which all study estimates were 100%, and proportion attending treatment of those referred to treatment (I^2^ = 59.1%).

**Fig. 2:**
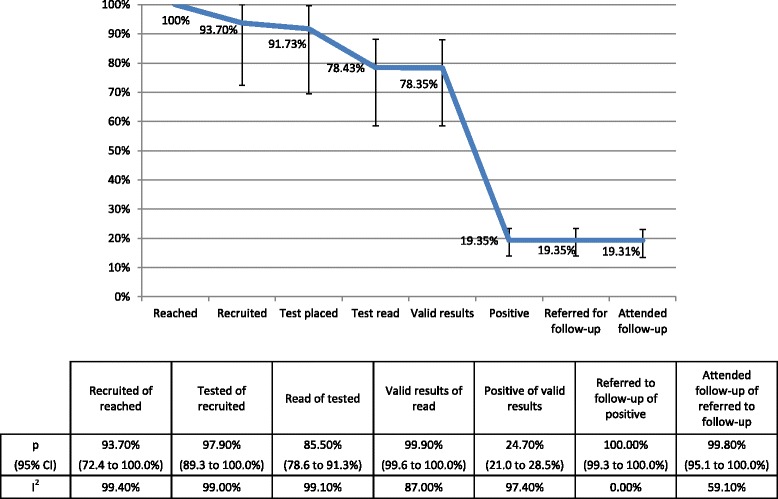
Cumulative proportion of homeless populations retained in each cascade step. TB targeted testing and linkage to care in the United States. TST-based studies only. Proportions retained from each step to the next are displayed in the table below

Adjacent step and cumulative proportions in the testing and linkage to care cascade are shown in Fig. 2. For a hypothetical cohort of 1000 homeless persons reached in studies using TST, we estimate that 784 (95% CI 585 to 880) would have valid test results, 194 would test positive (140 to 234), all 194 (139 to 234) would be referred to follow-up and 193 (134 to 231) would attend follow-up.

**Fig. 3:**
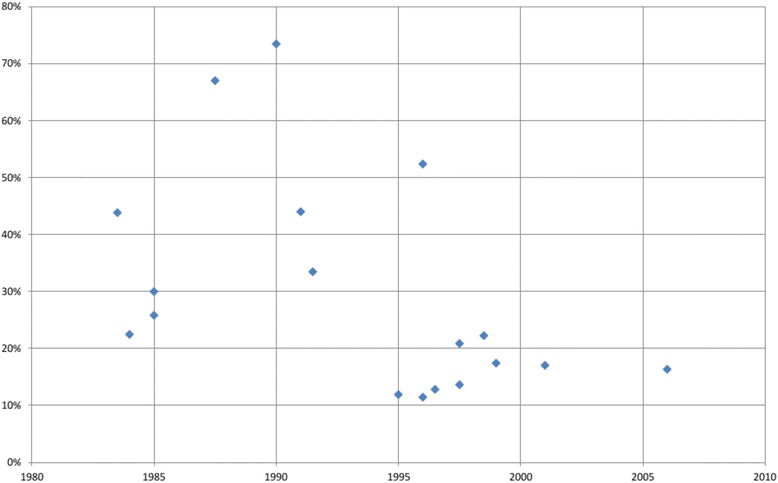
Proportions testing positive of those with valid results by year in homeless populations. TB targeted testing and linkage to care in the United States. TST-based studies only. Midpoint year of data collection was used when data collection accrued over multiple years

The proportions testing positive declined over time (Spearman’s ρ = − 0.57, *p* = 0.046). Figure 3 displays this finding graphically.

### Testing and linkage to care cascade – IGRA studies

Two studies that relied exclusively on IGRA [27, 28], recruited participants from health care facilities in San Francisco. Neither study reported numbers reached, recruited, or referred to follow-up. The pooled proportion of persons with valid test results of those tested was 97.0% (95% CI 96.8 to 97.3%) and the pooled proportion testing positive of those with valid test results was 8.9% (8.4 to 9.4%). One of these studies reported that 274 (67%) of 411 persons testing positive attended follow-up care [27].

One study [11] relied on a combination of TST and IGRA for testing without stratifying results by test type, but appears to have used mostly IGRA. In this study, of 1421 persons with valid test results, 185 (13%) tested positive, and 158 attended follow-up care.

### Recruitment type sub-analysis

Table 2 displays pooled proportions stratified by recruitment type for studies using TST for testing. Persons recruited at health care facilities (usually clinics for homeless persons) were retained at higher proportions for the first three steps of the cascade than persons recruited from other types of facilities. This difference is only statistically significant at α = 0.05 for recruited of reached.

**Table 2:** Proportions for select steps in TB targeted testing cascade among homeless persons, by recruitment method

Cascade Step	Number of studies*	N	Proportion	95% CI	Number of studies*	N	Proportion	95% CI	*P* value for difference between recruitment types
	Healthcare facility based				Other service based^**^				
Recruited of reached	1	829	100%	99.5 to 100%	1	750	78.1%	75.0 to 80.9%	< 0.001
Tests placed of recruited	2	923	100%	99.9 to 100%	5	2775	95.2%	80.8 to 100%	0.081
Valid results of tests placed	4	3926	93.9%	65.1 to 91.1%	6	26,839	78.6%	70.8 to 85.5%	0.247
Positive of valid results	4	3000	33.6%	18.0 to 51.2%	11	33,661	23.5%	19.1 to 28.3%	0.249
Referred to follow-up of positive	1	33	100%	89.6 to 100%	2	113	100%	98.3 to 100%	1.000
Attended follow-up of referred	1	33	100%	89.6 to 100%	2	113	100%	98.3 to 100%	1.000

^*^Not all studies reported data on all cascade steps

^**^Other service based recruitment includes recruitment from shelters, single room occupancy hotels, drop-in/day centers, substance abuse treatment facilities, meal programs, and other agencies that provide services to a primarily homeless or very low income clientele

## Discussion

This systematic review is, to the best of our knowledge, the first systematic review analyzing the performance of programs testing for LTBI, rather than TB disease, in homeless persons in the United States. We generally found promising yields of persons tested and LTBI detected. Compliance with testing was generally high, and even though some persons tested with TST did not return to have their tests read, we estimate that nearly four out of five persons reached by these targeted testing programs will obtain valid test results. More than 99% of persons who tested positive for infection were successfully referred to and attended at least one session of follow-up care. The proportion of persons testing TST positive of those with valid results (25%) is higher than would be expected among the general public [35]. In studies using TST, nearly 15% of persons who had tests placed did not return at the appropriate time to have them read. Because IGRA does not require a return visit to obtain test results, it may be possible to communicate test results when the patient makes contact with the testing organization at a later date, or over the phone or through mail for persons who have means to receive these types of communications, thus it is possible that use of IGRA will reduce the number of persons who remain unaware of their infection. Although the primary goal of these testing programs was to detect cases of LTBI, we found that an average of 1.2% of persons who tested positive were found to have TB disease on follow-up evaluation. Thus, LTBI targeted testing programs in homeless populations may also aid in the detection of cases of TB disease, which may lead to earlier diagnosis and less time spent with a transmissible infection.

We elected not to include data on treatment initiation and adherence as outcomes in this review, as we anticipated that most studies that included treatment outcomes would start with persons diagnosed with LTBI, rather than following a cohort through testing and diagnosis. Current recommended LTBI treatment options for most adults include shorter course regimens such as 4 months of daily rifampin, or 12 weekly doses (3 months) of isoniazid with rifapentine by directly observed therapy (DOT), in addition to the standard treatment of 9 months of daily or intermittent (by DOT) isoniazid [36]. Randomized controlled trials [37, 38], a meta-analysis [39], and an observational study [40] suggest that shorter course LTBI treatment regimens are associated with significantly higher treatment completion rates, even among persons experiencing homelessness and other marginalized groups. A systematic review of adherence to newer short-course regimens in persons experiencing homelessness would be a valuable companion piece to this review, and allow assessment of the entire LTBI continuum of care in this high risk population.

### Limitations

Meta-analyses often include a formal assessment of study bias. The Cochrane recommended tool for bias assessment was developed for the purpose of evaluating studies that compare an intervention with a control condition. This tool was considered inappropriate for the evaluation of this study because this analysis does not seek to compare one condition to another. We are unaware of any widely used and well accepted instrument for the evaluation of biases in studies that seek to estimate the yield of disease screening programs, therefore we did not conduct a formal bias assessment of individual studies. However, we can speculate on the types of biases that may have affected our results. A likely major source of bias is publication bias. The results of TTT programs for homeless persons are rarely published because assessments done by busy public health workers are intended only to inform the program. It is possible programs that publish or otherwise disseminate their results differ from programs that do not. For example, programs may publish about exceptionally successful programs, or conversely, publish about programs that faced exceptional challenges. Funnel plots of proportion with tests read of those with tests placed and proportions with positive tests of those with valid results for TST studies (Additional file 5) did not reveal any obvious asymmetry, but funnel plot asymmetry is largely due to the use of null-hypothesis significance testing. Because the included studies were not comparative in nature, there was no significance testing for the individual study proportions and publication bias may shift the entire distribution rather than causing asymmetry. Another consequence of incomplete reporting of targeted testing programs is that it is not possible to know what proportion of the homeless population is currently reached by these programs, which in turn makes it difficult to estimate the possible effects of scaling up testing in this population.

Misclassification is another potential source of bias; numbers of persons who were retained at each step of the cascade may not have been recorded correctly, and mistakes may have been made in interpreting test results. Studies did not generally provide sufficient evidence to evaluate the risk of bias due to misclassification.

In 2015, 66.4% of reported cases of TB disease in the United States were in persons who were born in another country, and TB incidence was nearly 13 times higher in persons born outside the US than those born in the US. Per data from the Online Tuberculosis Information System, 30.5% of recently homeless persons with TB diseases between 2011 and 2015 were also born outside the US [41]. However, we found very little information in our included studies regarding the intersection of these two critical risk populations in our included studies. Only one study stated the proportion of the study population that was born abroad; this study found that 17.3% of 260 homeless persons tested for TB in Los Angeles in the early 1990’s were non-US born [16].

There are also issues that limit the generalizability of our results. We included 17 studies conducted wholly or in part prior to 2000. The preponderance of older studies limits generalizability due to possible changes in LTBI prevalence. While the proportion of reported cases of TB disease in persons experiencing recent homelessness has remained at about 6% since the mid-1990s, the number of incident cases of TB disease in the United States have decreased in both the general public and homeless populations, with only 495 cases reported in 2015 in persons experiencing homelessness in the last year vs. 795 cases reported in 2005 [2]. The decline in cases in persons experiencing homelessness may be partially due to the active case-finding activities that have been advocated for these populations since the 1990’s [34]. We also found that studies were mainly conducted in coastal urban areas, meaning that our results may generalize poorly to testing programs for homeless persons in other settings.

Most studies in this review relied on TST, rather than IGRA for testing [42], and the studies that did use IGRA reported a limited number of cascade steps. In addition, the two studies that used IGRA exclusively were both conducted in San Francisco, and both recruited from healthcare facilities, limiting generalizability of results. Given these limitations, we do not think it is appropriate to use the results of this review to compare relative performance of TST with IGRA. The two studies which relied exclusively on IGRAs for testing did not report the number of persons (either those testing positive or overall) who received their test results, but in a separate review [unpublished] of targeted blood-borne infection testing for homeless persons, we estimated that only 57.9% of persons testing positive for HCV infection and 50.8% of persons testing positive for HIV infection were given test results. Additional studies are needed to assess LTBI test positivity and referral for follow-up care using the newer IGRA diagnostics.

Almost all of the studies included in this review recruited participants from health care facilities, shelters, or other types of service agencies. Only two [11, 16] included persons recruited through outreach or street venue based sampling. Because some homeless persons cannot or do not access services, most of the programs we reviewed likely left a large subpopulation of homeless persons without access to testing. We have conducted some stratified analyses by the type of service agency where recruitment was done, but failure to find a significant difference between proportions should be interpreted with caution because tests for heterogeneity are low-powered and frequently fail to reject false hypotheses.

We found high heterogeneity in most of our pooled proportions, indicating a high probability that study results differed from one another due to underlying program factors, rather than random variability. The random effects model that we used to calculate pooled proportions explicitly allows pooling of results sampled from differing underlying probability distributions. However, since our results indicate that real-world performance is likely to differ substantially between programs, program planners, modelers, and others who wish to incorporate our results into their work should consider our confidence intervals, in addition to our point estimates.

## Conclusions

CDC recommends TTT for populations at risk for TB, which is an important strategy for the United States to achieve TB elimination. Persons in congregate settings, including homeless shelters, are among populations at highest risk for TB transmission and outbreaks. In addition to identification of previously undiagnosed cases of TB among persons experiencing homelessness and prevention of TB outbreaks, TTT contributes to future TB prevention through treatment of LTBI. The U.S. Preventive Services Task Force issued a B grade recommendation that testing for LTBI in persons at increased risk for infection provides moderate benefit [43]. We found that 24.7% of homeless persons with valid TST results tested positive for TB infection, compared with estimated LTBI prevalence in the general population of 14.4% in 1971-72, 4.3% in 1999-2000, and 4.7% in 2011-2012 [35, 44]. These results support classification of homeless persons as a high risk population for TB infection. We also found that large proportions of targeted populations were successfully tested, and that most persons testing positive were successfully referred to follow-up care. TTT programs that use IGRAs for testing and shorter course LTBI treatment regimens may be promising ways to improve TB prevention among persons experiencing homelessness [38]. Further research is needed to reflect changes in LTBI prevalence among homeless persons over time, to evaluate the relative benefits of screening with TSTs vs IGRAs, and to synthesize the literature on treatment adherence in persons experiencing homelessness.

## Additional files

Additional file 1: Database search strategies and yield. (DOCX 19 kb)

Additional file 2: Data extraction sheet. (XLSX 56 kb)

Additional file 3: Articles screened at the full text level. (DOCX 32 kb)

Additional file 4: **Table S1.** Proportion and 95% confidence interval for homeless populations retained in TB targeted testing (for TST-based only) and linkage to care cascade in the United States. (DOCX 22 kb)

Additional file 5: Funnel plots. (PDF 71 kb)

## Abbreviations

CDC: Centers for Disease Control and Prevention
IGRA: Interferon-gamma release assay
LTBI: Latent tuberculosis infection
TB: Tuberculosis
TST: Tuberculin skin test
TTT: Targeted tuberculosis testing
