# Supplementary material for: Care Cascade for targeted tuberculosis testing and linkage to Care in Homeless Populations in the United States: a meta-analysis
Source: BMC Public Health. 2018 Apr 12;18:485. doi: 10.1186/s12889-018-5393-x (PMC5897923; doi:10.1186/s12889-018-5393-x)
Supplement: Supplementary file 1 — Database search strategies and yield. (DOCX 19 kb) [file 12889_2018_5393_MOESM1_ESM.docx]

# Additional file 1

# Database search strategies and yield (Search date: 13 June 2016)

Searched databases: PubMed, Embase, Cochrane Central Register, Web of Science

No date or language limits.

- Total: k=3,566
- Duplicates removed: k=915
- Records remaining: k=2651

| Search | PubMed Query | Items found |
| --- | --- | --- |
| #5 | Search #1 AND #2 AND #3 AND #4 | 1033 |
| #4 | Search (Tuberculosis[mh] OR HIV infections[mh] OR hepatitis b[mh] OR hepatitis c[mh] OR tuberculosis[tiab] OR TB[tiab] OR LTBI[tiab] OR HIV*[tiab] OR "human immunodeficiency virus"[tiab] OR "blood-borne”[tiab] OR bloodborne[tiab] OR “hepatitis b”[tiab] OR “hepatitis c”[tiab] OR HBV[tiab] OR HCV[tiab] OR “hep b”[tiab] OR “hep c”[tiab]) | 671627 |
| [#3](http://www-ncbi-nlm-nih-gov.ucsf.idm.oclc.org/pubmed/advanced) | Search (“Mass Screening”[mh] OR "Mobile Health Units"[Mesh] OR screen*[tiab] OR testing[tiab] OR program*[tiab] OR community-based[tiab] OR outreach[tiab] OR recruit*[tiab]) OR (target*[tiab] AND test*[tiab]) OR (peer[tiab] OR peer-based[tiab] OR chain-referral[tiab] OR snowball[tiab] OR sampling[tiab] OR time-location[tiab] OR respondent-driven[tiab] OR “social marketing” [tiab] OR “health fair” [tiab]) OR (engage[tiab] OR engaged[tiab] OR engagement[tiab] OR retention[tiab] OR retained[tiab] OR linked[tiab] OR linkage[tiab] OR linkages[tiab] OR referral[tiab] OR referred[tiab]) OR (incentives[tiab] OR enablers[tiab] OR “culturally adapted”[tiab] OR “culturally relevant”[tiab]) OR ("Population Surveillance"[mh] OR “prevalence studies”[mh] OR prevalence[tiab]) | 2979110 |
| #2 | Search “Homeless Persons”[mh] OR “Homeless Youth”[mh] OR Homeless[tiab] OR homelessness[tiab] OR shelter*[tiab] OR “transitional housing” [tiab] OR “single room occupancy” [tiab] OR unhoused[tiab] OR “unstably housed” [tiab] OR “unstable housing” [tiab] OR itinerant[tiab] OR “street people”[tiab] OR “street person”[tiab] OR “street youth” [tiab] OR “living rough”[tiab] OR transients[tiab] OR drifters[tiab] OR migrants[tiab] OR vagabonds[tiab] OR hoboes[tiab] OR veterans[tw] | 144288 |
| #1 | Search “United States”[tiab] “United States”[mh] OR USA[tiab] OR US[tiab] OR Alabama[tiab] OR Alaska[tiab] OR Arizona[tiab] OR Arkansas[tiab] OR California[tiab] OR Colorado[tiab] OR Connecticut[tiab] OR Delaware[tiab] OR Florida[tiab] OR Georgia[tiab] OR Hawaii[tiab] OR Idaho[tiab] OR Illinois[tiab] OR Indiana[tiab] OR Iowa[tiab] OR Kansas[tiab] OR Kentucky[tiab] OR Louisiana[tiab] OR Maine[tiab] OR Maryland[tiab] OR Massachusetts[tiab] OR Michigan[tiab] OR Minnesota[tiab] OR Mississippi[tiab] OR Missouri[tiab] OR Montana[tiab] OR Nebraska[tiab] OR Nevada[tiab] OR “New Hampshire”[tiab] OR “New Jersey”[tiab] OR “New Mexico”[tiab] OR “New York”[tiab] OR “North Carolina”[tiab] OR “North Dakota”[tiab] OR Ohio[tiab] OR Oklahoma[tiab] OR Oregon[tiab] OR Pennsylvania[tiab] OR “Rhode Island”[tiab] OR “South Carolina”[tiab] OR “South Dakota”[tiab] OR Tennessee[tiab] OR Texas[tiab] OR Utah[tiab] OR Vermont[tiab] OR Virginia[tiab] OR Washington[tiab] OR “West Virginia”[tiab] OR Wisconsin[tiab] OR Wyoming[tiab] OR American[tiab] | 940959 |

Embase strings 2, 3, 4: “Search as broadly as possible”; “explode” terms when possible to capture synonyms and sub-terms. Regional string #1: Apart from “explosion” of one term, which would capture studies conducted in all US states, other terms must appear in titles or abstract. Without this restriction, records with these terms in institutional affiliations would also have been captured.

| No. | Embase Query | Results |
| --- | --- | --- |
| #5 | #1 AND #2 AND #3 AND #4 | 1477 |
| #4 | homeless OR 'homelessness'/exp OR 'homelessness' OR shelter OR shelters OR 'transitional housing' OR 'single room occupancy' OR unhoused OR 'unstably housed' OR 'unstable housing' OR itinerant OR 'street people' OR 'street person' OR 'street youth' OR 'living rough' OR transients OR drifters OR vagabonds OR hoboes OR 'veterans'/exp OR veterans | 204623 |
| #3 | 'tuberculosis'/exp OR 'tuberculosis' OR ‘TB’ OR 'ltbi' OR 'hiv'/exp OR 'hiv' OR 'human immunodeficiency virus'/exp OR 'human immunodeficiency virus' OR 'hepatitis b'/exp OR 'hepatitis b' OR 'hepatitis c'/exp OR 'hepatitis c' OR 'hbv'/exp OR 'hbv' OR hcv OR 'hep b' OR 'hep c' OR 'blood-borne' OR 'bloodborne' | 863451 |
| #2 | 'mobile health units'/exp OR 'mobile health units' OR screen OR screened OR 'screening'/exp OR 'screening' OR (targeted AND testing) OR program OR 'community based' OR outreach OR recruit OR recruitment OR peer OR 'peer-based' OR 'chain-referral' OR snowball OR 'sampling'/exp OR 'sampling' OR 'time-location' OR 'respondent-driven' OR 'social marketing'/exp OR 'social marketing' OR 'health fair' OR engage OR engaged OR engagement OR retention OR retained OR linked OR linkage OR linkages OR 'referral'/exp OR 'referral' OR referred OR incentives OR enablers OR 'culturally adapted' OR 'culturally relevant' OR surveillance OR 'prevalence'/exp OR 'prevalence' | 4554030 |
| #1 | 'united states'/exp OR 'usa':ab,ti OR 'alabama':ab,ti OR 'alaska':ab,ti OR 'arizona':ab,ti OR 'arkansas':ab,ti OR 'california':ab,ti OR 'colorado':ab,ti OR 'connecticut':ab,ti OR 'delaware':ab,ti OR 'florida':ab,ti OR 'georgia':ab,ti OR 'hawaii':ab,ti OR 'idaho':ab,ti OR 'illinois':ab,ti OR 'indiana':ab,ti OR 'iowa':ab,ti OR 'kansas':ab,ti OR 'kentucky':ab,ti OR 'louisiana':ab,ti OR 'maine':ab,ti OR 'maryland':ab,ti OR 'massachusetts':ab,ti OR 'michigan':ab,ti OR 'minnesota':ab,ti OR 'mississippi':ab,ti OR 'missouri':ab,ti OR 'montana':ab,ti OR 'nebraska':ab,ti OR 'nevada':ab,ti OR 'new hampshire':ab,ti OR 'new jersey':ab,ti OR 'new mexico':ab,ti OR 'new york':ab,ti OR 'north carolina':ab,ti OR 'north dakota':ab,ti OR 'ohio':ab,ti OR 'oklahoma':ab,ti OR 'oregon':ab,ti OR 'pennsylvania':ab,ti OR 'rhode island':ab,ti OR 'south carolina':ab,ti OR 'south dakota':ab,ti OR 'tennessee':ab,ti OR 'texas':ab,ti OR 'utah':ab,ti OR 'vermont':ab,ti OR 'virginia':ab,ti OR 'washington':ab,ti OR 'west virginia':ab,ti OR 'wisconsin':ab,ti OR 'wyoming':ab,ti OR 'american':ab,ti | 2302954 |

Cochrane (k=56) and Web of Science (k=1000)

| Search | Query (titles, abstracts and keywords) |
| --- | --- |
| #5 | #1 AND #2 AND #3 AND #4 |
| #4 | homeless OR homelessness OR shelter OR shelters OR “transitional housing” OR “single room occupancy” OR unhoused OR “unstably housed” OR “unstable housing” OR itinerant OR “street people” OR “street person” OR “street youth” OR “living rough” OR transients OR drifters OR vagabonds OR hoboes OR veterans |
| #3 | tuberculosis OR TB OR ltbi OR hiv OR hiv/aids OR “human immunodeficiency virus” OR “hepatitis b” OR “hepatitis c” OR hbv OR hcv OR “hep b” OR “hep c” OR blood-borne OR bloodborne |
| #2 | “mobile health units” OR screen OR screened OR screening OR (targeted AND testing) OR program OR community based OR outreach OR recruit OR recruitment OR peer OR peer-based OR chain-referral OR snowball OR sampling OR time-location OR “respondent-driven” OR “social marketing” OR “health fair” OR engage OR engaged OR engagement OR retention OR retained OR linked OR linkage OR linkages OR referral OR referred OR incentives OR enablers OR “culturally adapted” OR “culturally relevant” OR surveillance OR prevalence |
| #1 | “united states” OR usa OR alabama OR alaska OR arizona OR arkansas OR california OR colorado OR connecticut OR delaware OR florida OR georgia OR hawaii OR idaho OR illinois OR indiana OR iowa OR kansas OR kentucky OR louisiana OR maine OR maryland OR massachusetts OR michigan OR minnesota OR mississippi OR missouri OR montana OR nebraska OR nevada OR “new Hampshire” OR “new jersey” OR “new mexico” OR “new York” OR “north Carolina” OR “north Dakota” OR ohio OR oklahoma OR oregon OR pennsylvania OR “rhode island” OR “south Carolina” OR “south Dakota” OR tennessee OR texas OR utah OR vermont OR virginia OR washington OR “west Virginia” OR wisconsin OR wyoming OR american |
