# Supplementary material for: Care Cascade for targeted tuberculosis testing and linkage to Care in Homeless Populations in the United States: a meta-analysis
Source: BMC Public Health. 2018 Apr 12;18:485. doi: 10.1186/s12889-018-5393-x (PMC5897923; doi:10.1186/s12889-018-5393-x)
Supplement: Supplementary file 3 — Articles screened at the full text level. (DOCX 32 kb) [file 12889_2018_5393_MOESM3_ESM.docx]

Additional file 3 Articles screened at the full text level

# Care Cascade for Targeted Tuberculosis Testing and Linkage to Care in Homeless Populations in the United States: Meta-analysis of program data

Contents

[Care Cascade for Targeted Tuberculosis Testing and Linkage to Care in Homeless Populations in the United States: Meta-analysis of program data 1](#_Toc478122111)

[Included (k=23) 1](#_Toc478122112)

[Excluded: Doesn't report one or both required outcomes (k=6) 3](#_Toc478122113)

[Excluded: Did not specifically target homeless (k=4) 4](#_Toc478122114)

[Excluded: Could not be located (k=1) 4](#_Toc478122115)

[Excluded: Duplicate data (k=3) 4](#_Toc478122116)

[Excluded: Narrative review (k=1) 5](#_Toc478122117)

[Excluded: Not a study (k=1) 5](#_Toc478122118)

[Excluded: Not a testing program (k=1) 5](#_Toc478122119)

[Excluded: Inconsistencies in data reported (k=1) 5](#_Toc478122120)

## Included (k=23)

#### Peer Reviewed Literature (k=19)

Barry MA, Wall C, Shirley L. Tuberculosis screening in Boston's homeless shelters. Public Health Reports. 1986;101(5):487-94.

Bock NN, Metzger BS, Tapia JR, Blumberg HM. A tuberculin screening and isoniazid preventive therapy program in an inner-city population. American journal of respiratory and critical care medicine. 1999;159(1):295-300.

Cheung RC, Hanson AK, Maganti K, Keeffe EB, Matsui SM. Viral hepatitis and other infectious diseases in a homeless population. Journal of Clinical Gastroenterology. 2002;34(4):476-80.

Dewan PK, Grinsdale J, Liska S, Wong E, Fallstad R, Kawamura LM. Feasibility, acceptability, and cost of tuberculosis testing by whole-blood interferon-gamma assay. BMC Infectious Diseases. 2006;6.

Falchook G, Gaffga C, Eve S, Ali J. Tuberculosis screening, referral, and treatment in an inner city homeless shelter in Orleans parish. The Journal of the Louisiana State Medical Society : official organ of the Louisiana State Medical Society. 2000;152(8):398-404.

Forman PD, Kinney C. Evaluation of a tuberculosis screening questionnaire for use in an Alaskan homeless population. Alaska medicine. 2003;45(4):94-101.

Gelberg L, Panarites CJ, Morgenstern H, Leake B, Andersen RM, Koegel P. Tuberculosis skin testing among homeless adults. Journal of General Internal Medicine. 1997;12(1):25-33.

Griffin RG, Hoff GL. Tuberculosis screening in Kansas City homeless shelters. Missouri medicine. 1999;96(10):496-9.

Kimerling ME, Shakes CF, Carlisle R, Lok KH, Benjamin WH, Dunlap NE. Spot sputum screening: evaluation of an intervention in two homeless shelters. The international journal of tuberculosis and lung disease : the official journal of the International Union against Tuberculosis and Lung Disease. 1999;3(7):613-9.

Kong PM, Tapy J, Calixto P, Burman WJ, Reves RR, Yang Z, et al. Skin-test screening and tuberculosis transmission among the homeless. Emerg Infect Dis. 2002;8(11):1280-4.

Lashley M. A targeted testing program for tuberculosis control and prevention among Baltimore City's homeless population. Public Health Nursing. 2007;24(1):34-9.

McAdam JM, Brickner PW, Scharer LL, Crocco JA, Duff AE. The spectrum of tuberculosis in a New York City men's shelter clinic (1982-1988). Chest. 1990;97(4):798-805.

McAdam JM, Bucher SJ, Brickner PW, Vincent RL, Lascher S. Latent tuberculosis and active tuberculosis disease rates among the homeless, New York, New York, USA, 1992-2006. Emerging Infectious Diseases. 2009;15(7):1109-11.

Morrow R, Fanta J, Kerlen S. Tuberculosis screening and anergy in a homeless population. The Journal of the American Board of Family Practice / American Board of Family Practice. 1997;10(1):1-5.

Neims SR, Fantry LE, Lee EU. Tuberculin positivity among the homeless. Journal of health care for the poor and underserved. 1992;3(2):263-9.

Nolan CM, Elarth AM, Barr H, Saeed AM, Risser DR. An Outbreak of Tuberculosis in a Shelter for Homeless Men - a Description of Its Evolution and Control. American Review of Respiratory Disease. 1991;143(2):257-61.

Paul EA, Lebowitz SM, Moore RE, Hoven CW, Bennett BA, Chen A. Nemesis revisited: tuberculosis infection in a New York City men's shelter. American journal of public health. 1993;83(12):1743-5.

Sakai J, Kim M, Shore J, Hepfer M. The risk of purified protein derivative positivity in homeless men with psychotic symptoms. Southern Medical Journal. 1998;91(4):345-8.

Torres RA, Mani S, Altholz J, Brickner PW. Human immunodeficiency virus infection among homeless men in a New York City shelter. Association with Mycobacterium tuberculosis infection. Archives of internal medicine. 1990;150(10):2030-6.

#### TB Notes and Conference Abstracts (k=4)

Alexander S. Engaging Community Stakeholders to Control a Homeless Shelter TB Outbreak in Indiana. TB Notes. 2011(4):7-10.

Higashi J. Programmatic Experience with IGRA Testing: San Francisco. North America Regional IUATLD Conference; March1, 2014; Boston, Massachusettes 2014.

Saez HV, Valencia ES, Susser E. Concurrent TB and HIV Prevalence among homeless men with mental illness in the latest TB epidemic in NYC. XIV International AIDS Conference; 7-12 July, 2002; Barcelona, Spain 2002.

Valencia ES, Saez H. Assessment of TB screening inclusion in time-limited case management for hard-to-reach severe mentally ill individuals at risk for HIV: TB screening and critical time intervention (CTI) in New York City. XV International AIDS Conference; 11-16 July, 2004; Bangkok, Thailand 2004.

## Excluded: Doesn't report one or both required outcomes (k=6)

Gelberg L, Doblin BH, Leake BD. Ambulatory health services provided to low-income and homeless adult patients in a major community health center. Journal of General Internal Medicine. 1996;11(3):156-62.

Gupta V, Sugg N, Butners M, Allen-White G, Molnar A. Tuberculosis among the homeless--preventing another outbreak through community action. The New England journal of medicine. 2015;372(16):1483-5.

Layton MC, Cantwell MF, Dorsinville GJ, Valway SE, Onorato IM, Frieden TR. Tuberculosis screening among homeless persons with AIDS living in single-room-occupancy hotels. American journal of public health. 1995;85(11):1556-9.

Mayo K, White S, Oates SK, Franklin F. Community collaboration: prevention and control of tuberculosis in a homeless shelter. Public health nursing (Boston, Mass). 1996;13(2):120-7.

Rendleman NJ. Mandated tuberculosis screening in a community of homeless people. American journal of preventive medicine. 1999;17(2):108-13.

Robertson MJ, Clark RA, Charlebois ED, Tulsky J, Long HL, Bangsberg DR, et al. HIV seroprevalence among homeless and marginally housed adults in San Francisco. American journal of public health. 2004;94(7):1207-17.

## Excluded: Did not specifically target homeless (k=4)

Alvarez S, Kasprzyk DR, Freundl M. Two-stage skin testing for tuberculosis in a domiciliary population. The American review of respiratory disease. 1987;136(5):1193-6.

Morano JP, Zelenev A, Walton MR, Bruce RD, Altice FL. Latent tuberculosis infection screening in foreign-born populations: a successful mobile clinic outreach model. American journal of public health. 2014;104(8):1508-15.

Schluger NW, Huberman R, Holzman R, Rom WN, Cohen DI. Screening for infection and disease as a tuberculosis control measure among indigents in New York City, 1994-1997. International Journal of Tuberculosis and Lung Disease. 1999;3(4):281-6.

Simon JA, McVicker SJ, Ferrell CR, Payne CB, Jr. Two-step tuberculin testing in a veterans domiciliary population. Southern Medical Journal. 1983;76(7):866-9, 72.

## Excluded: Could not be located (k=1)

Townsend MH, Stock MS, Morse EV, Simon PM. HIV, TB, and mental illness in a health clinic for the homeless. The Journal of the Louisiana State Medical Society : official organ of the Louisiana State Medical Society. 1996;148(6):267-70.

## Excluded: Duplicate data (k=3)

Gelberg L, Andersen RM, Leake BD. The Behavioral Model for Vulnerable Populations: application to medical care use and outcomes for homeless people. BMC Health Services Research. 2000;34(6):1273-302.

Saez H, Valencia E, Conover S, Susser E. Tuberculosis and HIV among mentally ill men in a New York City shelter. American journal of public health. 1996;86(9):1318-9.

White MC, Tulsky JP, Dawson C, Zolopa AR, Moss AR. Association between time homeless and perceived health status among the homeless in San Francisco. Journal of Community Health. 1997;22(4):271-82.

## Excluded: Narrative review (k=1)

Fusco M. Tuberculosis in New York City's homeless population: a public health nightmare. The Pharos of Alpha Omega Alpha-Honor Medical Society Alpha Omega Alpha. 1994;57(4):2-8.

## Excluded: Not a study (k=1)

Moss AR, Hahn JA, Tulsky JP, Daley CL, Small PM, Hopewell PC. Tuberculosis in the homeless. A prospective study. American journal of respiratory and critical care medicine. 2000;162(2 Pt 1):460-4.

## Excluded: Not a testing program (k=1)

Segal SP, Gomory T, Silverman CJ. Health status of homeless and marginally housed users of mental health self-help agencies. Health & social work. 1998;23(1):45-52.

## Excluded: Inconsistencies in data reported (k=1)

Glicksman R, Brickner PW, Edwards D. Tuberculosis screening and treatment of New York City homeless people. Annals of the New York Academy of Sciences. 1984;435:419-21.
