# Supplementary material for: Care Cascade for targeted tuberculosis testing and linkage to Care in Homeless Populations in the United States: a meta-analysis
Source: BMC Public Health. 2018 Apr 12;18:485. doi: 10.1186/s12889-018-5393-x (PMC5897923; doi:10.1186/s12889-018-5393-x)
Supplement: Supplementary file 4 — Table S1. Proportion and 95% confidence interval for homeless populations retained in TB targeted testing (for TST-based only) and linkage to care cascade in the United States. (DOCX 22 kb) [file 12889_2018_5393_MOESM4_ESM.docx]

# **Additional file 4**

Table S1. Proportion^[[1]](#footnote-1)^* and 95% confidence interval for homeless populations retained in TB targeted testing (for TST-based only) and linkage to care cascade in the United States

|  | **Recruited** | **Test placed** | **Test read** | **Valid results** | **Positive** | **Referred to follow-up** | **Attended follow-up^[[2]](#footnote-2)^†** |
| --- | --- | --- | --- | --- | --- | --- | --- |
| **Reached** | **93.7%^[[3]](#footnote-3)^**** | **88.0%** | **81.7%** | **77.4%** | **22.2%** | **34.5%** | **28.4%** |
|  | **(72.4 to 100%)** | **(34.5 to 100%)** | **(38.6 to 100.0%)** | **(34.0 to 99.9%)** | **(11.6 to 35.0%)** | **(29.1 to 40.1%)** | **(16.1 to 42.5%)** |
|  | **3 studies** | **3 studies** | **5 studies** | **5 studies** | **5 studies** | **2 studies** | **3 studies** |
| **Recruited** |  | **97.9%^**^** | **80.2%** | **79.4%** | **23.0%** | **10.7%** | **16.5%** |
|  |  | **(89.3 to 100%)** | **(60.2 to 94.4%)** | **(59.2 to 94.0%)** | **(14.7 to 32.4%)** | **(5.7 to 19.1%)** | **(13.1 to 20.1%)** |
|  |  | **8 studies** | **9 studies** | **9 studies** | **9 studies** | **1 study** | **2 studies** |
| **Test placed** |  |  | **85.5%^**^** | **81.3%** | **19.7%** | **23.4%** | **11.7%** |
|  |  |  | **(78.6 to 91.3%)** | **(74.1 to 87.6%)** | **(15.3 to 24.6%)** | **(17.2 to 30.1%)** | **(2.5 to 26.1%)** |
|  |  |  | **14 studies** | **14 studies** | **15 studies** | **2 studies** | **4 studies** |
| **Test read** |  |  |  | **99.9%^**^**  **(99.6 to 100.0%)**  **19 studies** | **24.3%**  **(20.6 to 28.1%)**  **19 studies** | **26.2%**  **(14.5 to 39.9%)**  **4 studies** | **21.3**  **(10.7 to 34.3%)**  **6 studies** |
| **Valid results** |  |  |  |  | **24.7%^**^** | **27.8%** | **20.4%** |
|  |  |  |  |  | **(21.0 to 28.5%)** | **(14.3 to 43.7%)** | **(10.3 to 32.7%)** |
|  |  |  |  |  | **20 studies** | **4 studies** | **7 studies** |
| **Positive** |  |  |  |  |  | **100.0%^**^** | **85.6%** |
|  |  |  |  |  |  | **(99.3 to 100.0%)** | **(61.5 to 99.5%)** |
|  |  |  |  |  |  | **4 studies** | **7 studies** |
| **Referred to follow-up** |  |  |  |  |  |  | **99.8%^**^** |
|  |  |  |  |  |  |  | **(95.1 to 100.0%)** |
|  |  |  |  |  |  |  | **4 studies** |
| Color reflects the # of studies pooled in reported proportion. | | |  | 1 study | 2 studies | 3-4 studies | 5+ studies |

1. * All proportions with two or more studies reporting data are pooled using random-effects. [↑](#footnote-ref-1)
2. † Number of persons who attend at least one session of further evaluation and treatment services. This can include people who did not start treatment or were judged to be poor candidates for treatment. [↑](#footnote-ref-2)
3. ** Cumulative proportions displayed in Figure 2 are products of these proportions. [↑](#footnote-ref-3)
