# Supplementary material for: Care Cascade for targeted tuberculosis testing and linkage to Care in Homeless Populations in the United States: a meta-analysis
Source: BMC Public Health. 2018 Apr 12;18:485. doi: 10.1186/s12889-018-5393-x (PMC5897923; doi:10.1186/s12889-018-5393-x)

Appendix E: Funnel plots for selected proportions, tuberculin skin test targeted testing among homeless populations in the United States (target line is random effects pooled estimate)

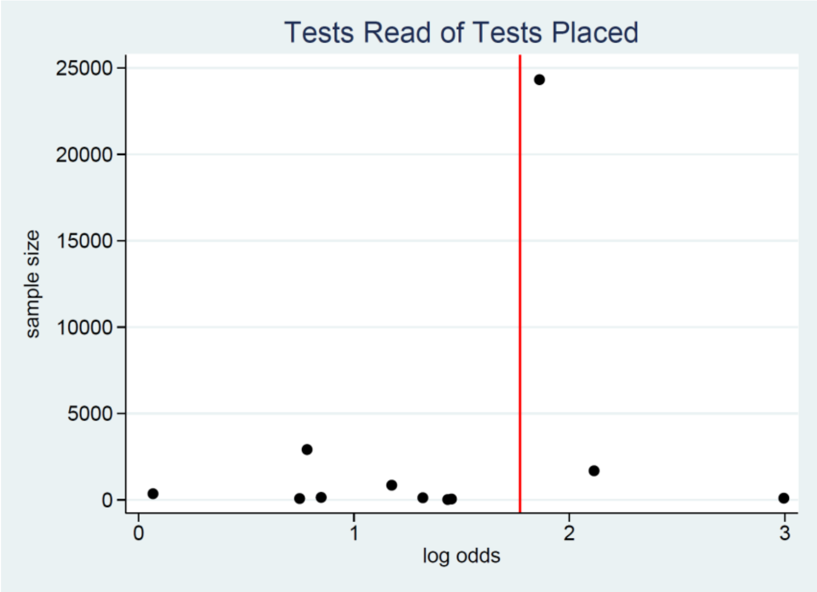

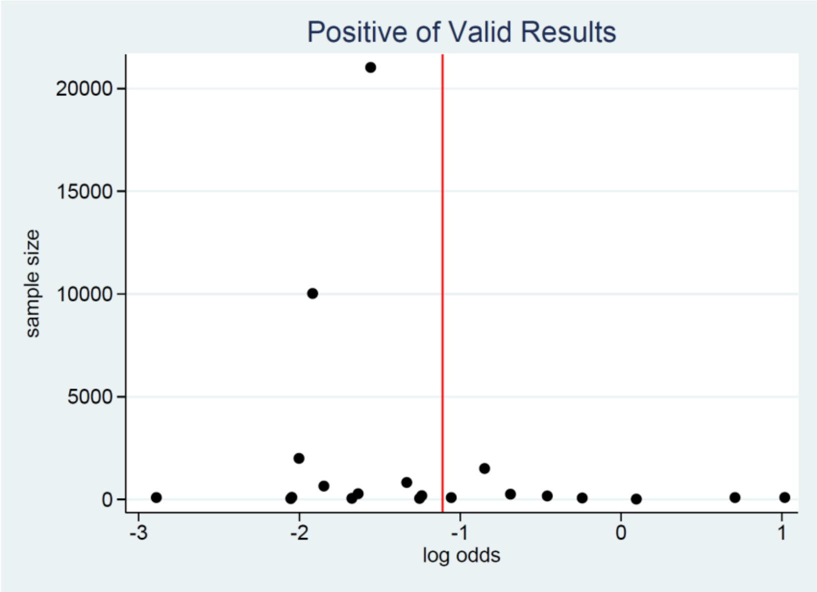

Supplement: Supplementary file 5 — Funnel plots. (PDF 71 kb) [file 12889_2018_5393_MOESM5_ESM.pdf]
